# Tricuspid Valve Replacement Using the Right Atrial Appendage Valve: Techniques and 1-Year Results

**DOI:** 10.1093/icvts/ivaf207

**Published:** 2025-09-15

**Authors:** Ahmad Ali Amirghofran, Seyed Alireza Mirhosseini, Roozbeh Narimani-Javid, Mohammad Reza Edraki, Mohammad Rafati Navaei, Hamid Mohammadi, Soorena Khorshidi, Salma Nozhat, Zahra Savand Roomi, Hamed Bazrafshan Driss, Sasan Shafiei, Alireza Arzhangzadeh

**Affiliations:** Cardiovascular Surgery Department, School of Medicine, Shiraz University of Medical Sciences, Zand Street, Shiraz 7134846114, Iran; Cardiovascular Research Center, Shiraz University of Medical Sciences, Zand Street, Shiraz 719365899, Iran; Department of Cardiology, Shiraz University of Medical Sciences, Shiraz 7164954937, Iran; Research Center for Advanced Technologies in Cardiovascular Medicine, Cardiovascular Diseases Research Institute, Tehran University of Medical Sciences, Tehran 1411713138, Iran; Pediatric Department, School of Medicine, Shiraz University of Medical Sciences, Shiraz 7193711351, Iran; Cardiovascular Surgery Department, Children’s Medical Center, Tehran University of Medical Sciences, Tehran 7991688115, Iran; Pediatric Department, School of Medicine, Shiraz University of Medical Sciences, Shiraz 7193711351, Iran; Department of Cardiology, Shiraz University of Medical Sciences, Shiraz 7164954937, Iran; Department of Cardiology, Shiraz University of Medical Sciences, Shiraz 7164954937, Iran; Department of Echocardiography, Abu Ali Sina Hospital, Shiraz 7199467985, Iran; Cardiovascular Research Center, Shiraz University of Medical Sciences, Zand Street, Shiraz 719365899, Iran; Department of Cardiology, Shiraz University of Medical Sciences, Shiraz 7164954937, Iran; Cardiovascular Research Center, Shiraz University of Medical Sciences, Zand Street, Shiraz 719365899, Iran; Department of Cardiology, Shiraz University of Medical Sciences, Shiraz 7164954937, Iran

**Keywords:** right atrial appendage valve, tricuspid valve replacement, tricuspid regurgitation

## Abstract

**Objectives:**

When tricuspid valve repair is unfeasible due to extensive damage or a complex congenital malformation, surgeons consider tricuspid valve replacement (TVR). However, it is still controversial and challenging to choose the best substitute. We aimed to introduce the innovative intraoperatively valve construction using the native right atrial appendage (RAA) tissue for TVR and investigate the short-term outcomes.

**Methods:**

This study recruited paediatric and adult patients with unrepairable severe tricuspid regurgitation (TR) who needed TVR. The patient’s RAA tissue was harvested and used to reconstruct a native bileaflet valve during surgery. Transthoracic echocardiography was conducted before operation, 6, and 12 months after surgery.

**Results:**

The procedure was successfully executed on 3 patients with valve destruction as a result of infective endocarditis, and 3 patients who had severe TR due to congenital anomalies. There was no mortality or related morbidity. All the constructed valves had proper function with no complications after the surgery. Follow-up echocardiographic studies showed stable and satisfactory valve function with no regurgitation or significant stenosis.

**Conclusions:**

The novel tricuspid valve operation using native RAA tissue demonstrates promising short-term results. Further studies with larger cohorts and longer follow-ups are required to confirm the technique’s reliability and long-term effectiveness.

## GRAPHICAL ABSTRACT

**Figure:**
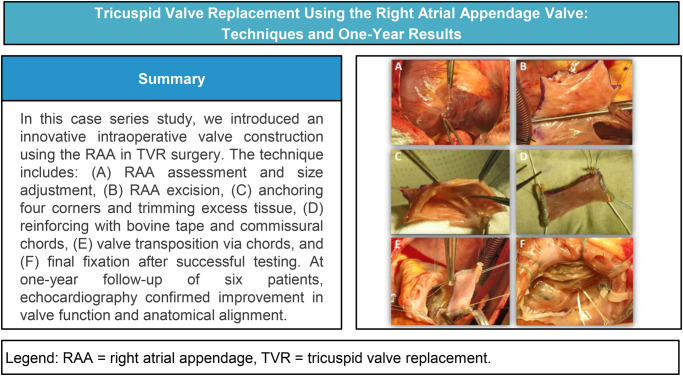

## INTRODUCTION

Tricuspid regurgitation (TR) is a significant valve disorder with limited data on management outcomes.^1^ Primary TR aetiologies include infective endocarditis (IE) (about 50%), followed by degenerative alterations, prolapse, and prosthetic valve failure.^2^ A growing interest in tricuspid valve (TV) repair has arisen from the understanding that untreated severe TR can lead to higher mortality.^3^^,^^4^ Surgery is recommended for symptomatic severe TR unresponsive to medical therapy.^1^^,^^5^ While right-side IE is usually managed conservatively, repair surgery can have promising outcomes.^6–8^

Tricuspid valve replacement (TVR) is considered only when repair is impossible due to extensive damage.^9^ Unsatisfactory outcomes of TVR may be attributed to late referrals with RV dysfunction and organ damage.^10^ Bioprosthetic valves are favoured in younger patients to avoid anticoagulation and immune responses.^11^ However, Mechanical valves offer greater durability and a reduced probability of reoperation.^12^ Allografts have shown promise, but graft choice depends on the patient’s condition.^13^^,^^14^

Transcatheter interventions are emerging but unsuitable for massive valve destruction or congenital anomalies like severe Epstein anomaly. A novel alternative is intraoperative native valve reconstruction using the patient’s right atrial appendage (RAA) tissue, which avoids anticoagulation and structural degeneration. We pioneered the use of the RAA valve for the right ventricular outflow tract in Tetralogy of Fallot (TOF) patients in 2018, with subsequent presentations of the technique and short to midterm results.^15^ Encouraged by these promising outcomes in more than 200 patients, we aimed to extend the application of the RAA valve for TVR, offering a hopeful prospect for the future of TVR.

## METHODS

### Study design and setting

This study was conducted at Faghihi and Dena Hospitals, evaluating TVR outcomes. Procedures were performed in a dedicated cardiovascular operating ward with postoperative care in the ICU. Surgical techniques and rationale were thoroughly explained to patients and their parents/guardians. All potential complications and risk prevention strategies were discussed, and written informed consent was obtained from all participants or their guardians, including consent for surgery and publication. The study followed the Declaration of Helsinki principles and received ethical approval from the Ethics Committee/Institutional Review Board of Shiraz University of Medical Sciences (ethics code IR.SUMS.MED.REC.1404.215). Data collection and storage adhered to the WMA Declaration of Taipei. The manuscript was prepared in line with the latest editorial guidelines for cardiothoracic surgery journals.^16^

### Participants

We screened both paediatric and adult patients in whom TV repair was deemed unfeasible. Eligible patients had severe TR resulting from either IE or congenital anomalies. Severe TR was defined as a regurgitant jet area exceeding 40% of the right atrium or a leaflet tethering distance greater than 8 mm. Patients with a preservable TV or multivalvular involvement due to IE or congenital anomalies affecting other valves or cardiac chambers were excluded.

### Data collection and patient care

Preoperative and postoperative TTE was performed to assess TV function, severity of TR, transvalvular pressure gradient, tricuspid annular plane systolic excursion, RV diameter, and the structure and function of other cardiac valves and chambers, following standard diagnostic protocols recommended by the American Society of Echocardiography and the European Association of Cardiovascular Imaging.^17^^,^^18^ Surgical, anaesthesia, perioperative data, and complications were recorded. IE patients were monitored for infection resolution, with postoperative ECG surveillance in the ICU and at follow-up. Patients received novel oral anticoagulants for 3 months, transitioning to aspirin. IE patients received vancomycin and meropenem until stabilization after surgery. In surgery, infected tissues were resected, and the RAA valve was constructed.

### Surgical technique

A detailed description of the RAA valve creation for use in the pulmonary position is available on CTSNet (https://www.ctsnet.org/article/how-make-valve-rvot-right-atrial-appendage), along with the technical video demonstrating its application in TVR (**Video 1**—https://www.ctsnet.org/article/tricuspid-valve-replacement-right-atrial-appendage-valve-first-report).

### Creating the appropriate size RAA valve

**Figure 1.**
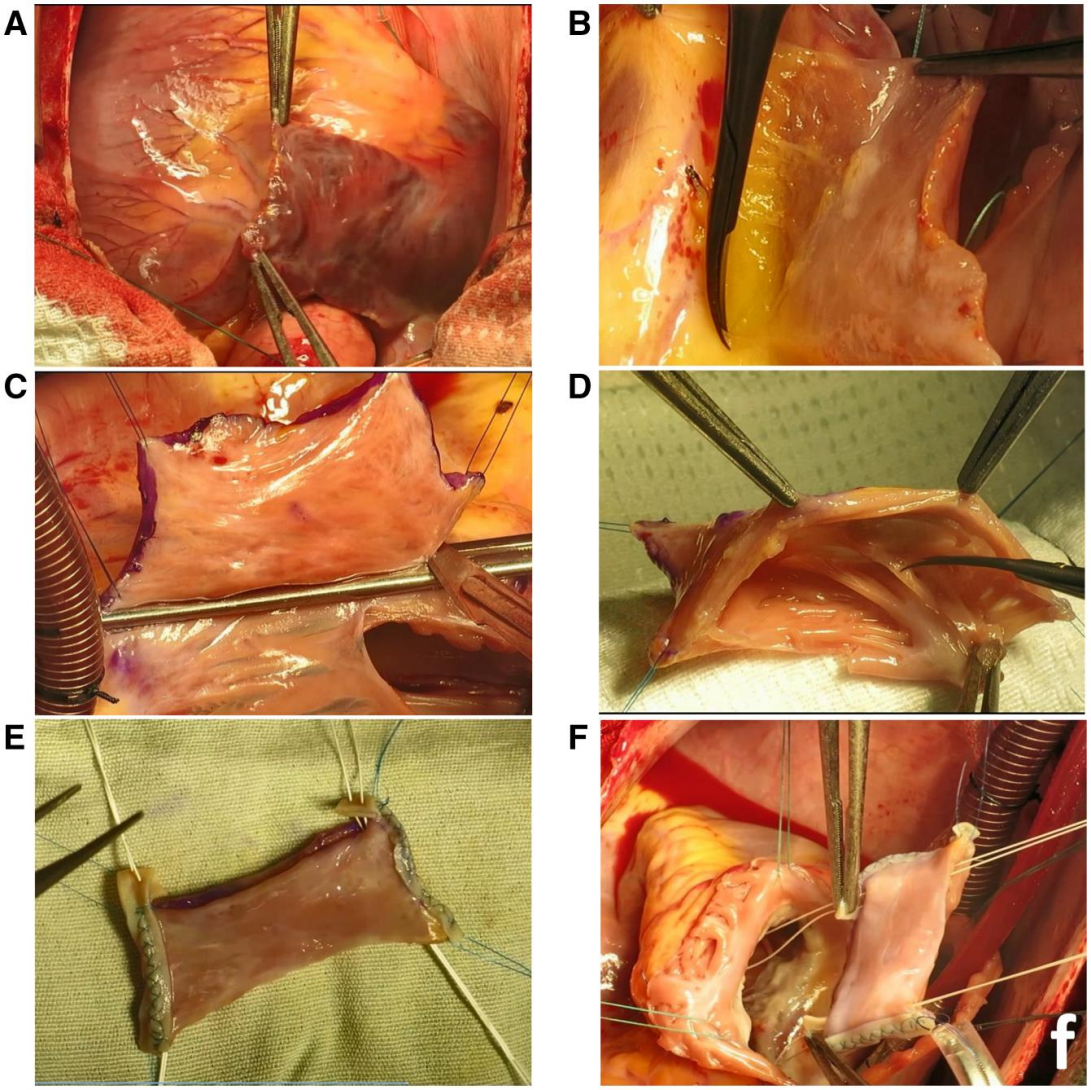
Right Atrial Appendage Valve Preparation for Tricuspid Valve Replacement Surgery. (A) evaluation of right atrial appendage. (B, C) Adjusting the valve size (height and width). (D) Preparing the valve after incision and size adjustment. (E) Strengthening commissures with bovine pericardium tape (3-4 mm, sutured in 2 rows) and passing chords through the patch. (F) Positioning the valve in the ventricle and attaching artificial chords

After midline sternotomy, the RAA is carefully evaluated before cardiopulmonary bypass (**Figure 1A**). The width of RAA, which is crucial in creating a fit-size valve, and the height of RAA are measured (eg, 40 × 12 mm in case 1). Direct superior and inferior vena-cava cannulation is performed. The left atrial appendage is considered if additional width is needed. The entire destructed tissue is excised. To ensure the proper structure of the valve with sufficient coaptation, we need a height of at least 70% of the width. So, we prepare a more extended height by releasing loose attachments of the appendage on the medial side (**Figure 1B**—eg, increasing from 12 to 30 mm in case 1). Then, we mark the borders of the appendage and cut the appendage over a cross-clamp at the base (**Figure 1C**). The atrial stamp is then closed in 2 layers. To prepare the valve, we fix the 4 corners of the appendage, and the inner large muscle bands are excised while smaller ones are left intact (**Figure 1D**). To increase the strength of the commissures to avoid later elongation and prolapse, they get the mechanical support from 3 to 4 mm of tape of bovine pericardium that is sutured in 2 rows (**Figure 1E**). The remaining 5 mm extra length of the bovine pericardium will be used later to connect artificial chords. The distal end of the appendage is then opened. The RAA valve is now ready to be replaced in its position.

### Annulus adjustment

We must adjust the annular dimension to accommodate the existing RAA valve before the transposition. For example, in case 1, a 40-mm annulus was reduced to 27 mm to match the RAA valve width. We achieved this reduction in size by pursuing the annulus, similar to the “De Vega” technique. Then, the sutures were tied over a 27-mm sizer to have both the RAA valve and annulus diameter of 27 mm.

### Transposition of the RAA valve

The valve is positioned with lateral wall of the appendage substituting the anterior leaflet. Two Teflon patches are sutured to the septum to support commissural artificial chords. CV-4 chords are passed through these platforms as U stitches. The chords are then passed through the extra commissural tape of the bovine patch and through inside the valve (**Figure 1E**). The valve is moved over these chords into the position in the ventricle (**Figure 1F**). The largest diameters of the annulus are marked at both sides and sutured to the proximal corners of the valve. Circular suturing of the proximal opening of the valve to the annulus is performed. This suture line may or may not be reinforced by a tape or bovine patch. The water test should be performed with the PA closed to determine the proper length of the artificial chords. We use the previously introduced adjustable pericardial lock technique by passing the chords through the small pieces of the pericardium twice (https://ctsnet.figshare.com/articles/media/Adjustable_Pericardial_Lock_Technique_for_Complex_Mitral_Valve_Repair/9955976/1?file=17929292). Then, the pericardial locks are slipped over the chords to the proper primary position where the commissures stay straight. Fine-tuning can be accomplished by further elongation or shortening the chords by moving the pericardial locks. Upon satisfactory testing, the artificial chords can be tied to the suitable position. If possible, we use a semirigid annuloplasty ring for proper annulus remodelling, better geometry, and long-term stability. The anterior leaflet should have more contribution in coaptation, and the coaptation line should be more posteriorly located.

## RESULTS

Six patients underwent TVR using the RAA valve: 3 with complete valve destruction due to IE (cases 1-3) and 3 with severe TR from congenital anomalies (cases 4-6). Ages ranged from 2 to 57 years and Operation times ranged from 5.5 to 6.5 hours. The patients baseline characteristics detailed in **Table 1**. Of the 3 IE cases, 2 patients (cases 2 and 3) were intravenous drug users with isolated TV involvement, while 1 patient (case 1) was a child with isolated TV endocarditis. Staphylococcus aureus was identified in all cases, and each received 8 weeks of intravenous meropenem and vancomycin therapy.

**Table 1.** Patients’ Baseline Characteristics

	Case 1	Case 2	Case 3	Case 4	Case 5	Case 6
Sex	Male	Male	Male	Female	Female	Male
Age (years)	14	51	37	3	12	58
Specific comorbidity	–	Hepatitis, intravenous drug abuser	Intravenous drug abuser	–	–	Severe asthma, interstitial lung disease
Signs and symptoms at admission	Chest pain, fever, chills, nausea, and vomiting	Lower extremity oedema	Chest pain, fever, chills, dyspnoea	Low O_2_ saturation, cyanosis	Dyspnoea	Dyspnoea, low O_2_ saturation
Diagnosis	Infective endocarditis	Infective endocarditis	Infective endocarditis	Ebstein’s anomaly	Congenital pathologic tricuspid valve	Ebstein’s anomaly
Lab data						
White blood cells ($\times 10^{9}/L$)	27.1	6.91	16.9	5.94	6.4	8.5
Haemoglobin (g/dL)	7.7	7.3	10.5	12.9	13.3	11.3
Platelet count ($\times 10^{9}/L$)	220	149	200	238	333	111
Blood urea nitrogen	30	60	17	16	10	13.2
Creatinine	0.9	1.7	0.6	0.48	0.5	0.89
CRP	>100	–	>100	<1	<1	<1
ESR	103	–	43	<1	<1	<1
Blood culture	No growth after 5 days	No growth after 5 days	No growth after 5 days	–	–	–
HBs Ag	Non-reactive	Non-reactive	Non-reactive	Non-reactive	Non-reactive	Non-reactive
HCV Ab	Non-reactive	Reactive	Non-reactive	Non-reactive	Non-reactive	Non-reactive
HIV Ag and Ab	Non-reactive	Non-reactive	Non-reactive	Non-reactive	Non-reactive	Non-reactive

Abbreviations: CRP = C-reactive protein; ESR = erythrocyte sedimentation rate; HBs Ag = hepatitis B surface antigen; HCV Ab = hepatitis C virus antibody; HIV Ag and Ab = human immunodeficiency virus antigen and antibody; O_2_ = oxygen.

**Figure 2.**
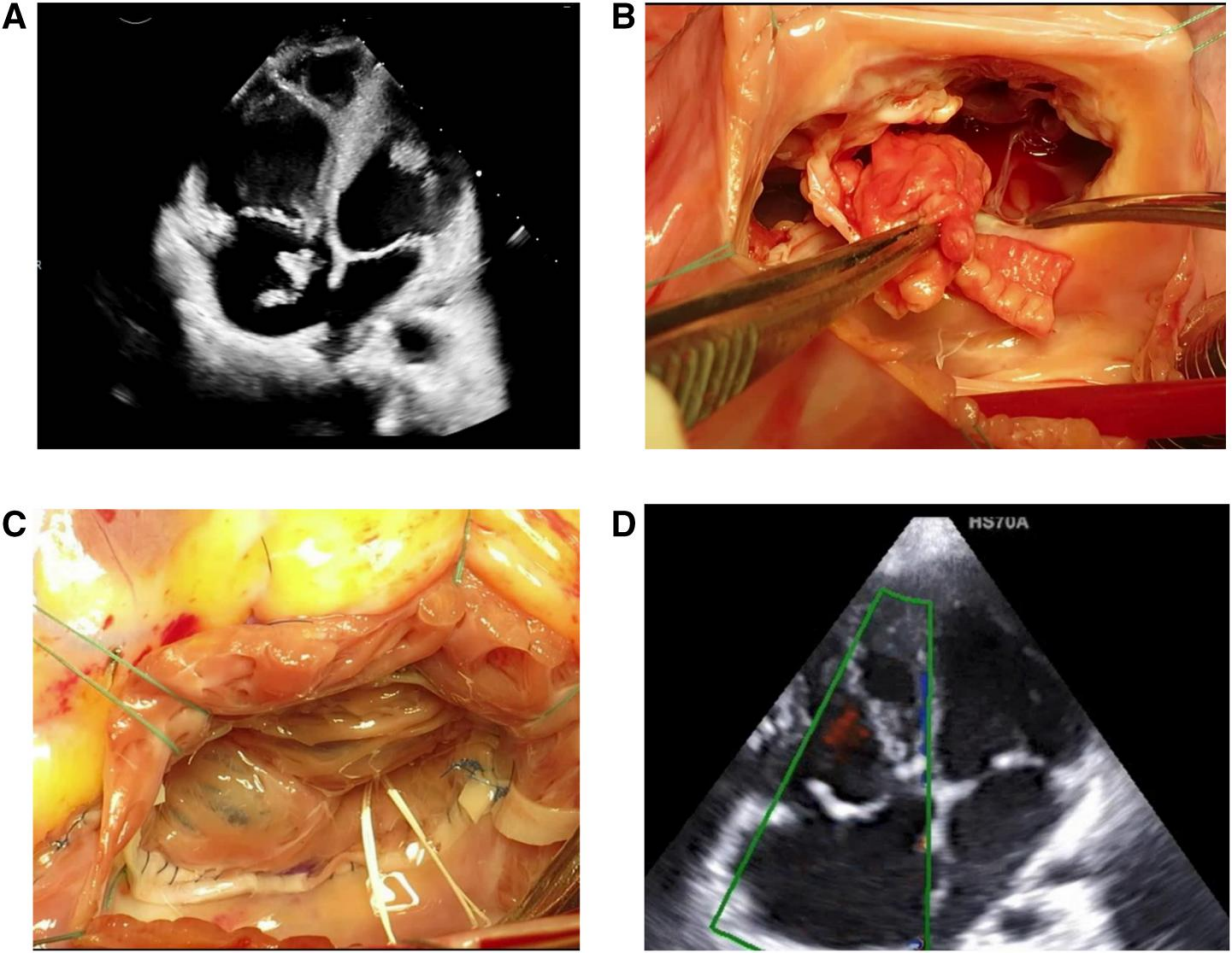
Right Atrial Appendage Valve Replacement for Tricuspid Valve Destruction Due to Infective Endocarditis. (A) Tricuspid valve destruction with large vegetation from infective endocarditis. (B) Multiple large vegetations leading to extensive leaflet destruction, making repair unfeasible. (C) Post-repair view showing well-coapted RAA valve with no regurgitation, using artificial chordae for positioning. (D) Reconstructed valve showing improved leaflet coaptation and satisfactory positioning, with no significant regurgitation on colour Doppler

Postoperative TTE showed normal TV function without complications. **Table 2** summarizes echocardiographic findings at baseline, 6 months, and 12 months. At the 12-month follow-up, the vegetations were healed, mild functional TS persisted, and the overall function and structure of the TV showed significant improvement. In cases 1 and 2, continued improvement was noted in TV and RV function. The echocardiographic and intraoperative views of TV in case 2 are illustrated in **Figure 2**. However, no data were available for case 2 at the 12-month follow-up as he left the addiction rehabilitation centre.

**Table 2.** Echocardiographic Findings Before Operation and 6-12 Months After Operation

Case	Tricuspid valve	Pulmonary valve	Right atrium	Right ventricle
Case 1—BO	Severe TR with a large, pedunculated, mobile vegetation (40 × 12 mm) extending from the RA to the RVOT. All 3 leaflets are involved. The anterior leaflet is predominantly affected with a ruptured chord at its tip. The septal leaflet has a small vegetation, while the posterior leaflet has a medium-sized vegetation and lacks coaptation with the anterior leaflet. All leaflets are motile.	Small suspicious vegetation, trivial PI.	NL^a^	NL
Case 1—6AO	No mass or vegetation observed. No significant turbulency in TV or RVOT. Septal TV motion mildly restricted; normal motion of the other 2 leaflets. Mild functional TS, TVPG = 6 mmHg, TVMG = 4 mmHg.	Mild PI, PVPG = 6 mmHg, PAP = 25 mmHg.	NL	Turbulency noted in mid RV at the site of artificial chordal attachment, without significant gradient. Normal RV size with lower limit of normal systolic function. RV diameter = 30 mm, TAPSE = 16 mm.
Case 1—12AO	No TR, mild functional TS, TVPG = 5 mmHg, TVMG = 3 mmHg.	Mild PI, PVPG = 6 mmHg, mean PAP = 28 mmHg.	NL	RV diameter = 32 mmHg, TAPSE = 20 mm.
Case 2—BO	Destructed TV with severe TR. A large multilobulated mobile mass (18 × 12 mm) on the anterior leaflet and a mobile mass (10 × 13 mm) on the posterior leaflet.	Mild PI.	Mildly dilated RA.	Dilated RV with normal systolic function.
Case 2—6AO	Mild TR, mild functional TS, no mobile mass or vegetation. TV leaflet motions are normal with normal thickness. TVPG = 7 mmHg, TVMG = 5 mmHg, TRG = 25 mmHg.	NL, PAP = 30-35 mmHg.	Mildly dilated RA.	No evidence of turbulency in the RV. RV diameter = 29 mm, TAPSE = 21 mm.
Case 2—12AO	NA	NA	NA	NA
Case 3—BO	Severe TR, large mass-like lesion (72 mm^2^), TRG = 17 mm.	NL	Mildly dilated RA.	Dilated with normal systolic function, TAPSE = 24 mm.
Case 3—6AO	No residual TR, small high-density mobile mass attached to the ventricular side of TV leaflets, likely a healed vegetation.	Mild to moderate PI.	NL	Dilated RV with moderate systolic dysfunction.
Case 3—12AO	TV ring annuloplasty in the appropriate position, artificial chordae visible in the RV with significant turbulency and increased gradient at the ring level, suggestive of functional TS. TVPG = 12 mmHg, TVMG = 8 mmHg. New TV leaflets mildly thick, no large mobile mass on artificial chordae.	NL, PVPG = 6 mmHg.	NL	RV diameter = 30 mm, mild RV systolic dysfunction.
Case 4—BO	Moderate to severe TR. ATVL is large and web-like (curtain) with 3 tethering sites and one fenestration, leading to 30 mmHg RVOT stenosis. PTVL is rudimentary. Septal leaflet is tethered (grade 1) with <5 mm free part and grade 1 displacement (10 mm). True TV annulus is 23 mm.	Trivial PI.	Dilated RA.	Dilated RV with good systolic function, TAPSE = 19 mm, No pulmonary hypertension.
Case 4—6AO	NL	NL	Dilated RA.	Mildly dilated RV.
Case 4—12AO	NL, TVMG = 7 mmHg.	Mild PI.	Dilated RA.	Dilated RV, TAPSE = 11 mm.
Case 5—BO	Congenital pathologic TV, tethered septal leaflet, proleptic anterior and posterior leaflets, and free TR. No Ebstein anomaly. TV annulus = 35-42 mm, peak gradient = 27 mmHg.	NL	Dilated RA.	Dilated RV, TAPSE = 22 mm, no pulmonary hypertension.
Case 5—6AO	NL, TVPG = 17 mmHg, TVMG = 7 mmHg.	NL, PVPG = 23 mmHg.	Dilated RA.	Dilated RV.
Case 5—12AO	NL, TVMG = 3 mmHg.	Mild PS, PVPG = 22 mmHg.	NL	NL, TAPSE = 7 mm.
Case 6—BO	Apical displacement of TV septal leaflet (30 mm) consistent with severe Ebstein anomaly. Significant tethering of anterior leaflet, severe TR. TRG = 37 mmHg, RVSP = 42 mmHg, TV annulus = 61 mm, TRVC = 6 mm.	Mild PI, mild pulmonary artery hypertension.	Severely dilated RA with large size.	Severely dilated RV with functional and atrialized RV due to TV septal leaflet displacement. TAPSE = 35 mm, severe RVOT dilation.
Case 6—6AO	NL	NL	Dilated RA.	Dilated RV.
Case 6—12AO	NL, TVPG = 8 mmHg, TVMG = 4 mmHg.	NL	Mildly dilated RA.	NL, TAPSE = 15 mm.

Abbreviations: 6AF, 6 months after operation; 12AF, 12 months after operation; AI, aortic insufficiency; ATVL, anterior tricuspid valve leaflet; BO, before operation; LV, left ventricle; MR, mitral regurgitation; NL, normal findings; PAP, pulmonary artery pressure; PFO, patent foramen ovale; PI, pulmonary insufficiency; PTVL, posterior tricuspid valve leaflet; PVPG, pulmonary tans-valvular peak pressure gradient; RA, right atrium; RV, right ventricle; RVOT, right ventricular outflow tract; RVSP, right ventricular systolic pressure; TAPSE, tricuspid annular plane systolic excursion; TR, tricuspid regurgitation; TRG, tricuspid regurgitation gradient; TRVC, tricuspid valve chordae; TVMG, tricuspid trans-valvular mean gradient; TVPG, tricuspid trans-valvular peak pressure gradient; TVR, tricuspid valve repair; TS, tricuspid stenosis.

a “NL” findings in the cardiac chambers indicate normal size, diameter, and function without any abnormalities, while in valve descriptions, they denote the absence of regurgitation, stenosis, or structural abnormalities.

**Figure 3.**
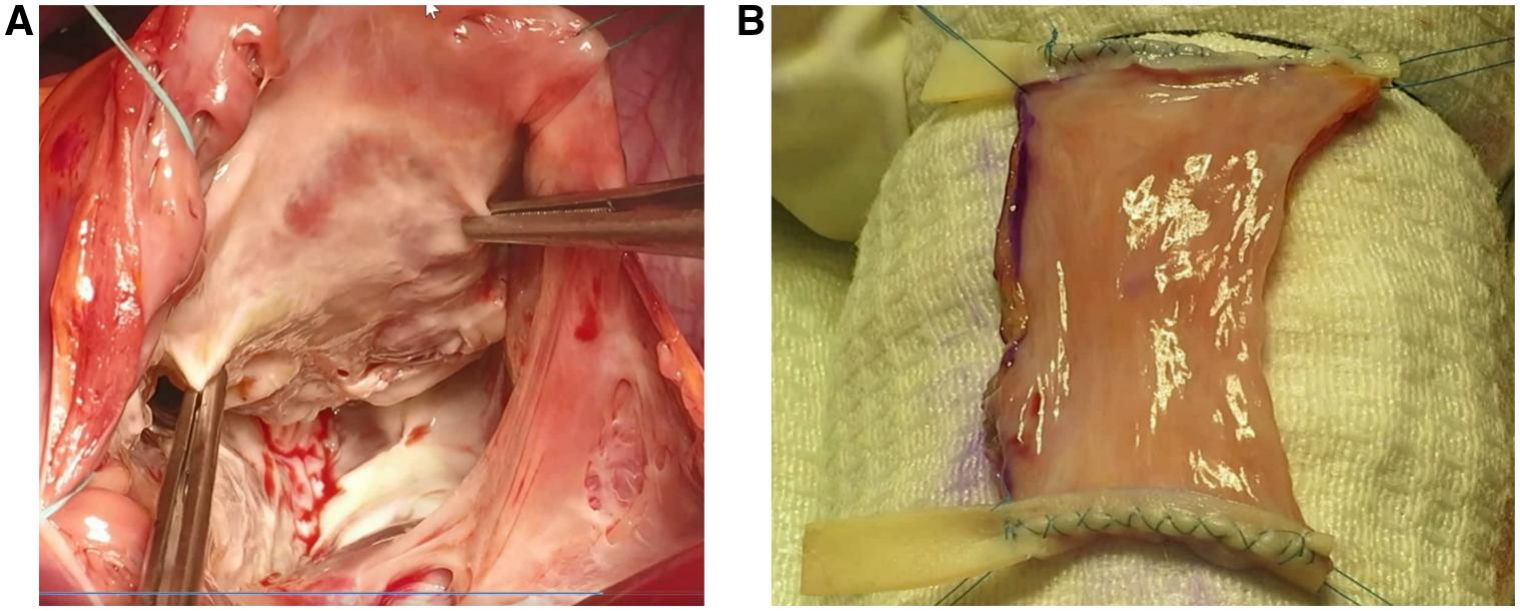
Severe Ebstein’s Anomaly Case. (A) Intraoperative view showing severe Ebstein’s anomaly with absent septal and posterior leaflets and a narrow anterior leaflet, making valve repair unfeasible. (B) Right atrial appendage valve prepared for implantation, with 2 bovine pericardial strips serving as scaffolds for anchoring the valve within the right ventricle

Among congenital cases, 2 patients (cases 4 and 6) were suffering from Ebstein’s anomaly. These cases were both type 3 Carpentier classification, with 2.5 and 4 cm displacement of the valve. The anterior leaflet was narrow in case 6 with severe tethering. In the other one (case 4), the anterior leaflet was short and fenestrated, without any chordal attachment in the midpart. Moreover, the posterior and septal leaflets were small and displaced in these cases (**Figure 3**). Based on these findings, they were not suitable for Cone repair. Case 5 was A non-Ebstein 12-year-old girl, exhibited congenital thick TV leaflets, tethered septal leaflet, and proleptic anterior and posterior leaflets (especially anterior leaflet) with free TR due to non-coaptation of leaflets. The thickened and retracted leaflets in this case had very short fibrotic cords restricting the leaflet motion, causing moderate TS and Severe TR. Twelve-month follow-up TTE in all 3 congenital cases demonstrated appropriate adaptation of the RAA valve and significant improvement in valve structure and overall cardiac function.

## DISCUSSION

In this study, we introduced an innovative technique for TVR using the RAA valve, which yielded positive short- and midterm outcomes in 6 patients. Several procedures for TV repair have been developed, each with its advantages and disadvantages. For limited IE, TV repair is preferred over TVR to avoid prosthetic valve deterioration and recurrent IE, especially in IV drug users.^19^ Similarly, repair techniques for severe TR due to Ebstein’s anomaly have shown acceptable results, but TVR is necessary in cases of extensive valve damage.

Historically, mechanical valves were favoured, but bioprostheses now make up over 80% of TVRs due to anticoagulation concerns.^20^ However, biological valve degeneration, particularly in younger patients, remains a limitation. RAA tissue remains viable after repositioning, with potential for growth. In previous studies, we demonstrated the effectiveness of RAA valves in TOF, with no degeneration or calcification to date.^15^ Building on positive midterm outcomes in over 200 patients, we extended the RAA valve technique to TVR. Due to structural differences between pulmonic and TVs, commissures in the TV position require suspension to ventricular muscles with artificial chords. Chordal length is crucial to prevent prolapse, and the pericardial lock technique is used to adjust this length, resembling the successful cylinder technique.^21^

**Figure 4.**
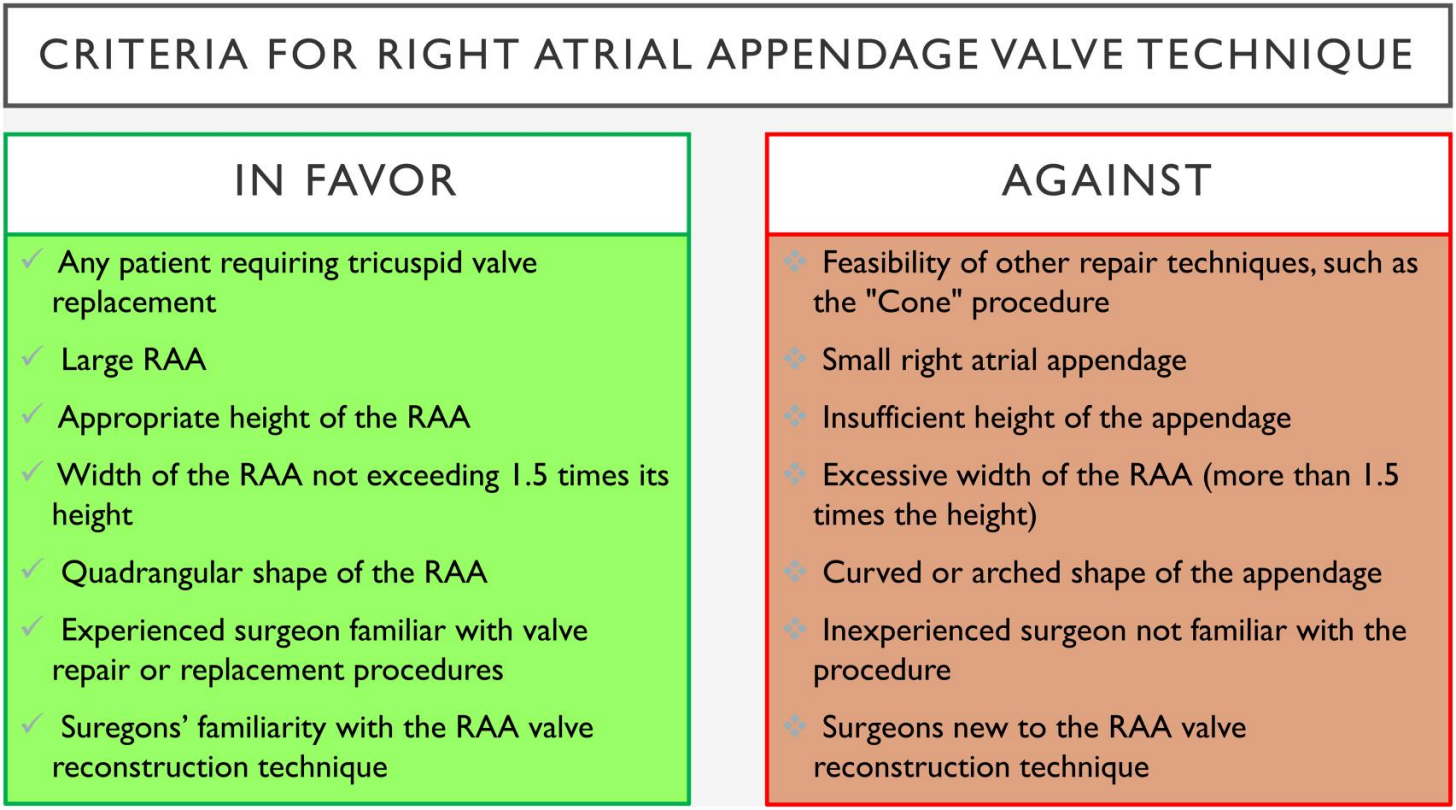
Criteria for Right Atrial Appendage Valve Technique. Abbreviation: RAA, right atrial appendage

The Cone procedure, introduced by da Silva in 2007, is the standard surgical approach for Ebstein’s anomaly, restoring normal valve anatomy and function by mobilizing the malformed leaflets and reattaching them in a cone-like configuration at the true annulus. It uses only native tissue, making it ideal for younger patients requiring a growing valve. Studies show excellent valve competence, low reoperation rates, and good right heart function after Cone repair.^22^^,^^23^ Prior approaches like the Carpentier or Hetzer repairs, focused on the anterior leaflet or partial repairs, often left patients with residual regurgitation and less durable results.^24^ In critically ill infants, techniques like the Knott-Craig repair or valve replacement were sometimes necessary but had long-term limitations.^25^ Compared to those earlier approaches, the Cone repair has now become the standard approach in experienced centers.^23^^,^^26^ The RAA valve technique offers a simplified, fully biological alternative, avoiding prosthetic materials and complex mobilization. It preserves right heart geometry, requires less reconstruction, and is particularly useful when leaflet tissue is inadequate or damaged. The RAA valve technique can be applied to any patient requiring TVR, with the ability to adjust the annulus size. However, an appropriately sized RAA with sufficient height and width is essential. The RAA should have a quadrangular shape, not a curved shape, and its width should be no more than 1.5 times its height. Fortunately, most patients with severe TR have an enlarged right atrium, providing a sufficiently large RAA. The surgeon must be experienced in valve repair or replacement and familiar with the innovative RAA valve technique. Key criteria for applying this technique are summarized in **Figure 4**.

### Limitations

The study’s limitations include the small sample size and short follow-up duration. Additionally, the technique was limited to patients with isolated TV involvement from IE or congenital anomalies, leaving its efficacy in multivalvular dysfunction or additional anomalies unknown. We used TTE, which, although widely available and practical, is operator dependent. Future studies incorporating cardiac magnetic resonance imaging should provide more accurate assessments of cardiac dimensions and valve performance.

## CONCLUSION

Tricuspid valve replacement using the RAA valve technique has shown promising results in terms of functional outcomes, utilization of native tissue, and the technical aspects of the procedure during a 1-year follow-up in cases where traditional repair options were not feasible. However, longer follow-up with a larger patient cohort is necessary to fully assess the long-term reliability and effectiveness of this technique.

## Data Availability

All relevant data are within the manuscript.
